# Optimization of Machining Parameters for Milling Zirconia Ceramics by Polycrystalline Diamond Tool

**DOI:** 10.3390/ma15010208

**Published:** 2021-12-28

**Authors:** Xuefeng Yan, Shuliang Dong, Xianzhun Li, Zhonglin Zhao, Shuling Dong, Libao An

**Affiliations:** 1 College of Mechanical Engineering, North China University of Science and Technology, No. 21 Bohai Road, Caofeidian Xincheng, Tangshan 063210, China; yanxf1226@163.com (X.Y.); lxzhl3109@163.com (X.L.); zzl18031566021@163.com (Z.Z.); 2 Shanxi Limin Industrial Co. Ltd., Jinzhong 030812, China; 3 Kama Automobile Company, No. 5888, Donghuan Road, Shouguang 262700, China; dsl15266367517@163.com

**Keywords:** zirconia ceramics, polycrystalline diamond tool, milling, finite element simulation

## Abstract

Zirconia ceramics are widely used in many fields because of their excellent physical and mechanical properties. However, there are some challenges to machine zirconia ceramics with high processing efficiency. In order to optimize parameters for milling zirconia ceramics by polycrystalline diamond tool, finite element method was used to simulate machining process based on Johnson-Cook constitutive model. The effects of spindle speed, feed rate, radial and axial cutting depth on cutting force, tool flank wear and material removal rate were investigated. The results of the simulation experiment were analyzed and optimized by the response surface method. The optimal parameter combination was obtained when the spindle speed, feed rate, radial and axial cutting depth were 8000 r/min, 90.65 mm/min, 0.10 mm and 1.37 mm, respectively. Under these conditions, the cutting force was 234.81 N, the tool flank wear was 33.40 μm when the milling length was 60 mm and the material removal rate was 44.65 mm^3^/min.

## 1. Introduction

Zirconia ceramics are characterized by high toughness, high bending strength, high wear resistance, excellent heat insulation, well corrosion resistance and biocompatibility [1,2,3], which have been expansively used in many areas such as aerospace, precision machinery and biomedicine [4,5,6,7,8]. High-efficiency processing for zirconia ceramics has become a research hotspot. The milling of zirconia ceramics can obtain complex three-dimensional structures and surface quality equivalent to grinding, which can make up for the limitations of existing processing technology to a certain extent [9]. However, it is different to machine zirconia ceramics because of their high toughness, high bending strength, high wear resistance and excellent heat insulation which cause severe tool wear and tipping, low surface quality and machining efficiency [10]. Polycrystalline diamond (PCD) tool has the advantages of high hardness, good thermal conductivity, low friction coefficient and low thermal expansion coefficient, which is an ideal tool for milling zirconia ceramics [11,12]. In recent years, a lot of research about surface quality, tool wear and material removal rate has been done when zirconia ceramics are machined by PCD tool [13,14,15].

Eleonora et al. [16] investigated the effects of cutting parameters on surface quality and tool wear cutting parameters on high speed hard cutting with PCD tool. The results showed that the material was mainly removed by combining ductile-brittle phases, and the tool wear was largely produced by workpiece material sticked to the tool. Bian et al. [17] studied the relationship between cutting parameters and brittle-tough critical cutting thickness during milling zirconia ceramics by using PCD tool. It was found that the appropriate increasing axial depth of cut can prevent the brittleness damage from affecting the machined surface and increase the material removal rate with the stabilization of the surface roughness within a certain range. Rong et al. [18] considered the PCD tool with bigger particle size possesses longer tool life. The surface roughness was primarily affected by feed per tooth, which increased with the increase of feed per tooth teeth. Wan et al. [19] established a thermal-mechanical coupling simulation model of zirconia grinding to simulate the subsurface damage. Under the Thermal-mechanical coupling effect, simulation results were deviation of less than 6% compared with the experimental results. Li et al. [20] investigated the temperature distribution influenced by temperature dependent thermal properties and heat flux profile based on a heat transfer model. The results shown that the temperature had the greatest impact by cutting depth. Xue et al. [21] built a finite element model to analyze the influence of depth of cut on stress changes and crack distribution during cutting process of zirconia ceramics. The results showed that the simulation analysis was beneficial for the optimization of machining parameters. Deng et al. [22] simulated the process of diamond fly-cutting zirconia ceramics, and studied the influence of tool geometric parameters and cutting parameters on stress distribution, brittle-plastic transition depth, cutting force and chip morphology. Hence, numerical simulation could be used to reveal the processing mechanism of milling zirconia ceramics.

During the milling of zirconia ceramic, the processing parameters should be accurately controlled because of the brittle fracture of zirconia ceramics, especially when the workpiece material is thin. Meanwhile, maching is accompanied by severe tool wear and low machining efficiency. It is very important to optimize the milling parameters of zirconia ceramics. In this paper, a finite element simulation model of PCD tool milling zirconia ceramics based on Johnson-Cook constitutive model was established. The milling experiments were designed with the central composite design method, and the simulation data was analyzed by regression analysis. The response surface method was used to analyze the effect of cutting parameters on cutting force, tool flank wear and material removal rate. The optimized parameter combination was obtained for the cutting force, tool flank wear, and material removal. The specific experiments were performed to verify simulation results.

## 2. Simulation Details

### 2.1. Constitutive Model

The Johnson-Cook constitutive equation was used to establish the constitutive model of zirconia ceramics milled by PCD tool. The model reflected the coupling effects of strain hardening, strain rate strengthening, and thermal softening during the cutting process. The expression is as follows [23]:

(1) $$\overset{¯}{\sigma }=\left[A+B{\left(\overset{¯}{\varepsilon }\right)}^{n}\right]\left[1+C\ln\left(\dot{\overset{¯}{\varepsilon }}/{\dot{\overset{¯}{\varepsilon }}}_{0}\right)\right]\left[1-{\left(\left(T-T_{r}\right)/\left(T_{m}-T_{r}\right)\right)}^{m}\right]$$

where, $\overset{¯}{\sigma }$ is equivalent flow stress (Mpa). *A*, *B*, *C*, *n*, *m* are the constants of the material under reference conditions, denoting yield stress (Mpa), strain hardening constant (Mpa), strengthening coefficient of strain rate, strain hardening coefficient and thermal softening coefficient, respectively. $\overset{¯}{\varepsilon }$ is equivalent plastic strain; $\dot{\overset{¯}{\varepsilon }}$ is equivalent plastic strain rate, and ${\dot{\overset{¯}{\varepsilon }}}_{0}$ is reference strain rate. *T*, *T_r_* and *T_m_* are maximum temperature of material, room temperature and melting temperature, respectively, usually measured in °C.

Johnson-Cook constitutive model parameters for zirconia ceramics are shown in Table 1.

### 2.2. Three-Dimensional Finite Element Model

**Figure 1:**
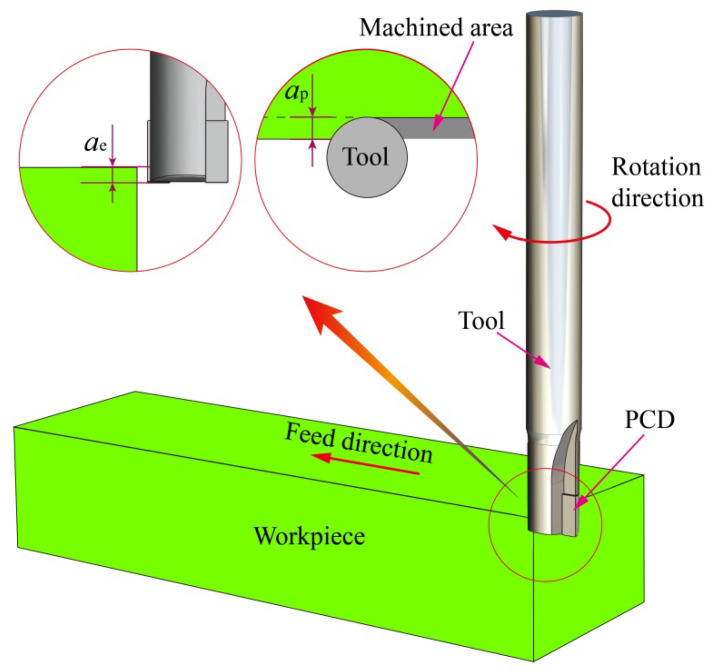
Milling schematic diagram.

The physical properties of zirconia ceramics and PCD are shown in Table 2. The PCD tool is a second straight-tooth groove end mill with a diameter of 8 mm, helix angle and rake angle of 0°, and rear angle of 10°. The size of the zirconia ceramic is 100 × 30 × 20 mm^3^. The coefficient of friction is 0.3 incorporating a modified coulomb friction law with dry milling [24]. The zirconia ceramic and PCD are adopted an 8-node hexahedral element (C3D8RT) and 4-node tetrahedral unit (C3D4T), respectively. The milling schematic diagram is shown in Figure 1.

The material removal rate *Q* is determined by the distance of milling and axial and radial depth per unit time. The *Q* is calculated by the equation:

(2) $$Q=v_{f}\alpha_{e}\alpha_{p}$$

where, $v_{f}$ is feed rate of tool feed rate, mm/s. $\alpha_{e}$ and $\alpha_{p}$ are the radial and axial depth of milling, mm, respectively.

**Figure 2:**
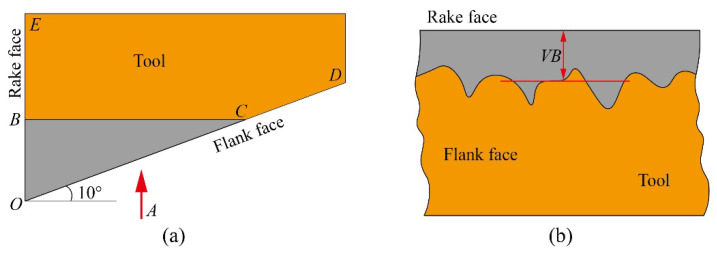
Schematic illustration of tool wear. (**a**) Tool wear cross section, (**b**) A-direction view.

During zirconia ceramic milled by PCD tool, the large cutting force will intensify the friction between the tool and the workpiece contact surface leading to severe damage on the tool surface, especially flank face. The rake and flank angle of the PCD tool used in this research is 0° and 10°, respectively. The schematic illustration of tool wear is shown in Figure 2. EOD is the shape of the tool. After the tool wear, the shape of the tool is EBCD as shown in Figure 2a,b shows the A-direction view of the tool. *VB* is the average wear of the flank face. In order to simplify the measurement of tool wear, tool wear in this research was replaced by *VB* [25].

Response surface method was used to design the simulation experiment, which could obtain the influence of experiment parameter on results and its significance. Combined with engineering experience, four cutting parameters with five different levels of each were studied in the simulation experiment. The factors level of spindle speed (*n*), feed rate (*v*_f_), radial depth of cut (*a*_e_), and axial depth of cut (*a*_p_) are shown in Table 3. The cutting force (*F*), tool flank wear (*VB*) and material removal rate (*Q*) were as the response performance indicator.

## 3. Results and Discussion

### 3.1. Simulation Results

**Figure 3:**
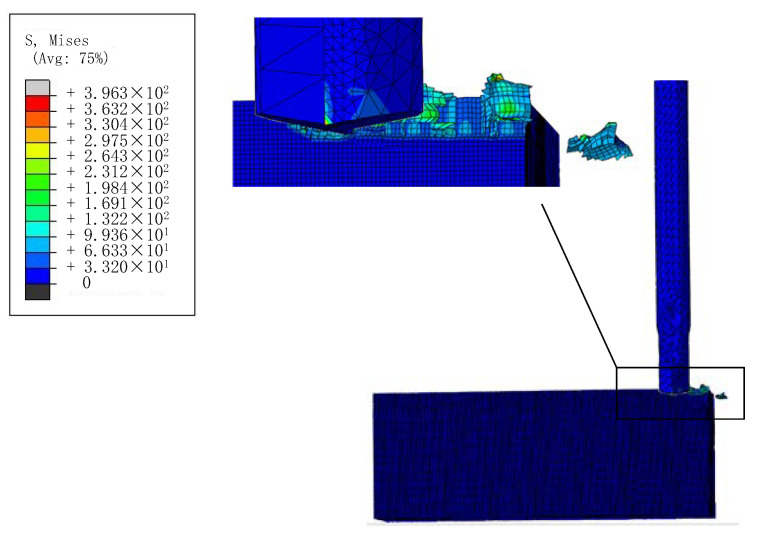
Schematic diagram of simulation results.

The simulation results of milling zirconia ceramics by PCD tool is shown in Figure 3. It can be seen that the stress mainly concentrated in the tip. For the simulation experiments, the simulation results of *F*, *VB* and *Q* under different *n*, *v*_f_, *a*_e_ and *a*_p_ with the milling length of 60 mm are shown in Table 4.

### 3.2. Response Surface Analysis

**Figure 4:**
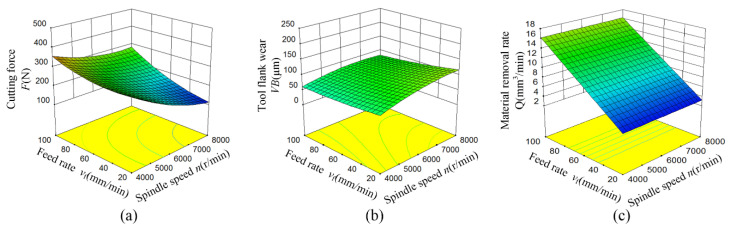
Response surface of spindle speed and feed rate on cutting force, tool flank wear and material removal rate. (**a**) Cutting force *F*, (**b**) Tool flank wear *VB*, (**c**) Material removal rate *Q*.

The influences of spindle speed and feed rate on cutting force, tool flank wear and material removal rate are shown in Figure 4. It can be seen that the cutting force decreases significantly with the increase of spindle speed as shown in Figure 4a. The reason for this is that the increase of spindle speed causing the temperature of the processing area rise which reduces the strength and hardness of zirconia ceramics. However, the influences of spindle speed on tool flank wear and material removal rate are not obvious as shown in Figure 4b,c. The cutting force and material removal rate are increased with the increase of feed rate, especially material removal rate. Increasing the feed rate could increase the scan area of the tool in unit time results in an increase in material removal rate. The interaction between spindle speed and feed rate has the most significant impact on the cutting force, followed by tool flank wear, but no significant impact on the material removal rate.

**Figure 5:**
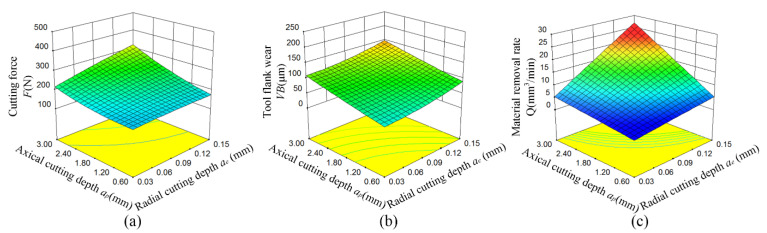
Response surface of radial depth of cut and axial depth of cut on cutting force, tool flank wear and material removal rate. (**a**) Cutting force *F*, (**b**) Tool flank wear *VB*, (**c**) Material removal rate *Q*.

The influences of radial and axial depth of cut on the cutting force, tool flank wear and material removal rate are shown in Figure 5. Radial depth of cut increase caused a slight increase of cutting force and tool flank wear, mainly because the cutting distance becomes longer and the cutting amount increases when the axial depth of the tool contact remains unchanged. The contact area between the tool and the workpiece increased with the increase of axial depth of cut [26]. Therefore, the cutting force and tool flank wear increased more than increasing the radial depth of cut as shown in Figure 5a–c shows that the material removal rate increased significantly regardless of whether the radial or axial depth of cut increased. The interaction between radial and axial depth of cut has a significant impact on the material removal rate, followed by cutting force and tool flank wear.

### 3.3. Parameter Optimization

Multiple regression fitting was used to analyze the influence of n, *v*_f_, *a*_e_ and *a*_p_ on *F*, *VB* and *Q*. The second-order regression prediction models of *F* (N), *VB* (mm) and *Q* (mm^3^/min) are shown as follow:

(3) $$\begin{array}{cc} F= & 906.66-0.13n-4.31v_{f}-2395.69a_{e}-137.59a_{p}+6.71\times 10^{-6}n^{2}+0.02v_{f}^{2}-4187.85a_{e}^{2}\\ & +38.41a_{p}^{2}+3.48\times 10^{-4}nv_{f}+0.28na_{e}-0.01na_{p}+0.92v_{f}a_{e}+0.84v_{f}a_{p}+1300.73a_{e}a_{p} \end{array}$$

(4) $$\begin{array}{cc} VB= & -224.65+0.12n-0.48v_{f}-1751.28a_{e}-57.01a_{p}-1.03\times 10^{-5}n^{2}+0.01v_{f}^{2}+8481.71a_{e}^{2}\\ & +4.28a_{p}{}^{2}-2.69\times 10^{-4}nv_{f}+0.09na_{e}+0.02na_{p}+5.851v_{f}a_{e}-0.13v_{f}a_{p}+1.60a_{e}a_{p} \end{array}$$

(5) $$\begin{array}{cc} Q= & 3.5\times 10^{5}-38.88n-3888v_{f}-2.59\times 10^{6}a_{e}-1.3\times 10^{5}a_{p}+0.32nv_{f}+216na_{e}\\ & +10.8na_{p}+21600v_{f}a_{e}+1080v_{f}a_{p}+7.2\times 10^{5}a_{e}a_{p} \end{array}$$

**Figure 6:**
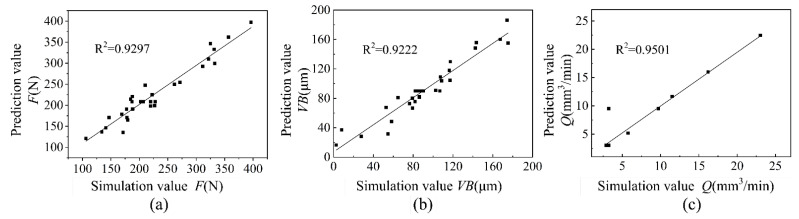
The relation between predicted and simulated values. (**a**) Cutting force *F*, (**b**) Tool flank wear *VB*, (**c**) Material removal rate *Q*.

Residual error was used to estimate whether the regression model is reasonable. Figure 6 shows the relation between predicted and simulated values of *F*, *VB* and *Q*. It can be seen that all sample points are close to a straight line, and there are no out-of-range sample points. The correlation coefficient (R^2^) of *F*, *VB* and *Q* is 0.9297, 0.9222 and 0.9501, respectively, which indicates that the second-order regression prediction models have less error and higher reliability [27,28].

In order to further analyze the experimental factors on *F*, *VB* and *Q*, the regression prediction models were analyzed by variance analysis. The results are shown in Table 5.

The F-value in Table 5 represents the ratio of the mean square between each group to the mean square within the group. If α is 0.05, the value of F_0.05_(14,15) is 2.42 according to F distribution table. The F-value of *F*, *VB* and *Q* is 14.17, 12.70 and 743.85, respectively, which is more than 2.42 indicating the prediction model established significance. Simultaneously, the *p*-values of the model are less than 0.05, which also shows the model is effective [29].

The *p*-values of *n*, *v*_f_, *a*_e_ and *a*_p_ in the *F* regression model are less than 0.001, showing that the four experimental factors have extremely significant effects on the cutting force. The *p*-value of *a*_e_*a*_p_ is 0.0033 < 0.05 indicating *a*_e_ and *a*_p_ with significant interactive effects on *F*. The F-values of *n*, *v*_f_, *a*_e_ and *a*_p_ are 34.56, 27.00, 26.54 and 80.76, respectively. According to the F-values, the influence of the four experimental factors on *F* is *a*_p_ > *n* > *v*_f_ >*a*_e_.

The *n*, *v*_f_, *a*_e_ and *a*_p_ have extremely significant effects on the *VB* because of the *p*-values of *n*, *a*_e_ and *a*_p_ in the *VB* regression model less than 0.001. The *p*-value of *na*_p_ is 0.0301 < 0.05, showing that spindle speed and axial depth of cut have a significant interactive effect on the *VB*. According to the F-values, the influence of the four experimental factors on *VB* is *a*_p_ > *a*_e_ > *n* > *v*_f_.

The *p*-values of *v*_f_, *a*_e_ and *a*_p_ in the *Q* regression model are less than 0.0001, which indicates *v*_f_, *a*_e_ and *a*_p_ have extremely significant effects on the material removal rate. The F-values of *n*, *v*_f_, *a*_e_ and *a*_p_ are 1.042, 2308.5, 2308.5 and 2308.5, respectively. According to the size of the data, the influence of the four experimental factors on *Q* is *v*_f =_ *a*_e =_ *a*_p_ > *n*.

In order to obtain multi-objective optimal machining parameters, the regression prediction models of *F*, *VB* and *Q* were considered comprehensively under the same weight. A set of optimal machining parameters with the smallest cutting force, the smallest tool flank wear, and the largest material removal rate were obtained: 8000 r/min for *n*, 90.65 mm/min for *v*_f_, 0.10 mm for *a*_e_, and 1.37 mm for *a*_p_. Under this condition, the *F* is 234.81 N, the *VB* is 33.40 μm, and the *Q* is 44.65 mm^3^/min under the milling length of 60 mm.

### 3.4. Model Validation with Experiments

In order to verify the validity of the prediction models, the experiments of milling zirconia ceramics by PCD tool were carried out in vertical drilling and tapping center TC500R. The experiments were repeated three times under the conditions of the optimal combination of machining parameters to obtain an average value. The results are shown in Table 6. According to the results of three experiments, the average values of *F*, *VB* and *Q* are 208.08 N, 29.24 μm, and 41.87 mm^3^/min, respectively. Compared with the predicted results, the relative errors of *F*, *VB* and *Q* are 11.38%, 12.46% and 6.23%, respectively, all less than 15%, which indicates that it is reasonable and feasible to use response surface method to optimize the machining parameters of milling zirconia ceramics by PCD tool.

## 4. Conclusions

In this paper, we established a finite element model to simulated milling zirconia ceramics by PCD tool. The influence of *n*, *v*_f_, *a*_e_ and *a*_p_ on *F*, *VB* and *Q* were studied. The response surface method was used to analyze and optimize the milling parameters. The second-order regression prediction models of *F*, *VB* and *Q* were established with the confidence level of each prediction model higher than 0.92. The influence of experimental factors on *F*, *VB* and *Q* is *a*_p_ > *n* > *v*_f_ >*a*_e_, *a*_p_ > *a*_e_ > *n* > *v*_f_ and *v*_f_ = *a*_e_ = *a*_p_ > *n*, respectively. When the multi-objective optimal machining parameters with *F*, *VB* and *Q* were under the same weight, the optimal parameters of *n*, *v*_f_, *a*_e_ and *a*_p_ are 8000 r/min, 90.65 mm/min, 0.10 mm, and 1.37 mm, respectively. Under this condition, *F* was 234.81 N, *VB* was 33.40 μm and *Q* was 44.65 mm^3^/min, when the milling length was 60 mm. Comparing the experimental and simulation results, the relative errors of *F*, *VB* and *Q* are 11.38, 12.46 and 6.23%, respectively. They are all smaller than 15% indicating that it is reasonable and feasible to use the response surface method to optimize the machining parameters of milling zirconia ceramics by PCD tool.

## Figures and Tables

**Table 1:** Johnson-Cook constitutive model parameters for zirconia ceramics [22].

*A*/MPa	*B*/MPa	*C*	*n*	*m*	*T_r_*/°C	*T_m_*/°C
930	310	0	0.6	0.6	25	1725

**Table 2:** Physical properties of workpiece and tool.

Material	Elastic Modulus E/(Pa)	Poisson’s Ratio μ	Thermal Conductivity κ/(W/m·K)	Heat Capacity c/(J/kg·K)	Density ρ/(kg/m^3^)
Zirconia ceramics	2.39 × 10^11^	0.3	2.6	400	6050
PCD	1.2 × 10^12^	0.2	1500	471.5	3520

**Table 3:** Test factors level.

No.	Control Factors	Level				
		−2	−1	0	1	2
1	*n*/(r/min)	4000	5000	6000	7000	8000
2	*v*_f_/(mm/min)	20	40	60	80	100
3	*a*_e_/(mm)	0.03	0.06	0.09	0.12	0.15
4	*a*_p_/(mm)	0.6	1.2	1.8	2.4	3.0

There are four factors, according to central composite design, so the numbers of corner points are 16. The total number of experiments was 30.

**Table 4:** Simulation results of zirconia ceramic milling.

No.	*n*/(r/min)	*v*_f_/(mm/min)	*a*_e_/(mm)	*a*_p_/(mm)	*F*/(N)	*VB*/(μm)	*Q*/(mm^3^/min)
1	5000	80	0.12	2.4	396.29	107.31	23.04
2	4000	60	0.09	1.8	332.62	2.70	9.72
3	5000	40	0.06	2.4	210.37	102.73	5.76
4	6000	60	0.09	0.6	179.58	8.25	3.24
5	7000	80	0.12	1.2	219.75	79.39	11.52
6	5000	40	0.12	1.2	177.62	81.92	5.76
7	6000	60	0.09	1.8	202.43	89.44	3.24
8	7000	40	0.12	1.2	141.08	116.97	5.76
9	8000	60	0.09	1.8	146.79	86.31	9.72
10	7000	40	0.06	1.2	106.08	79.43	2.88
11	6000	100	0.09	1.8	311.32	86.18	16.2
12	7000	40	0.06	2.4	171.49	142.52	5.76
13	7000	80	0.12	2.4	324.96	143.52	23.04
14	6000	20	0.09	1.8	169.13	117.32	3.24
15	7000	40	0.12	2.4	271.42	174.30	11.52
16	7000	80	0.06	2.4	222.31	108.54	11.52
17	6000	60	0.15	1.8	261.54	167.51	16.2
18	6000	60	0.09	1.8	219.73	86.45	9.72
19	6000	60	0.03	1.8	134.03	64.75	3.24
20	6000	60	0.09	1.8	205.13	106.89	9.72
21	7000	80	0.06	1.2	178.28	27.84	5.76
22	6000	60	0.09	1.8	187.19	90.34	9.72
23	5000	80	0.06	1.2	187.86	54.75	5.76
24	5000	80	0.12	1.2	184.71	76.46	11.52
25	6000	60	0.09	1.8	227.94	82.34	9.72
26	6000	60	0.09	3.0	356.75	175.33	16.2
27	5000	80	0.06	2.4	321.53	52.85	11.52
28	6000	60	0.09	1.8	206.95	84.90	9.72
29	5000	40	0.12	2.4	331.63	116.29	11.52
30	5000	40	0.06	1.2	226.95	58.30	2.88

**Table 5:** Analysis of variance of regression prediction models.

Source	*F*					*VB*					*Q*				
	Sum of Squares	df	Sum of Squares	df	Mean Square	F-Value	*p*-Value	Mean Square	F-Value	*p*-Value	Sum of Squares	df	Mean Square	F-Value	*p*-Value
Model	143,000	14	48,094.43	14	3435.32	12.70	<0.0001	3435.32	12.70	<0.0001	811.81	10	81.18	743.85	<0.0001
n	24,913.15	1	6309.58	1	6309.58	23.33	0.0002	6309.58	23.33	0.0002	0.00	1	0.00	1.04	1.0000
$v_{f}$	19,461.52	1	3372.04	1	3372.04	12.47	0.0030	3372.04	12.47	0.0030	251.94	1	251.94	2308.50	<0.0001
$a_{e}$	19,131.47	1	9389.17	1	9389.17	34.71	<0.0001	9389.17	34.71	<0.0001	251.94	1	251.94	2308.50	<0.0001
$a_{p}$	58,214.49	1	20,849.44	1	20,849.44	77.08	<0.0001	20,849.44	77.08	<0.0001	251.94	1	251.94	2308.50	<0.0001
$nv_{f}$	775.76	1	462.90	1	462.90	1.71	0.2105	462.90	1.71	0.2105					
$na_{e}$	1147.69	1	112.47	1	112.47	0.42	0.5288	112.47	0.42	0.5288					
$na_{p}$	1184.91	1	1552.36	1	1552.36	5.74	0.0301	1552.36	5.74	0.0301					
$v_{f}a_{e}$	4.92	1	196.84	1	196.84	0.73	0.4070	196.84	0.73	0.4070		1			
$v_{f}a_{p}$	1626.31	1	40.01	1	40.01	0.15	0.7059	40.01	0.15	0.7059					
$a_{e}a_{p}$	8770.79	1	0.01	1	0.01	0.00	0.9945	0.01	0.00	0.9945					
$n^{2}$	1235.29	1	2897.50	1	2897.50	10.70	0.0052	2897.50	10.70	0.0052					
$v_{f}{}^{2}$	1283.61	1	445.42	1	445.42	1.65	0.2189	445.42	1.65	0.2189					
$a_{e}{}^{2}$	389.65	1	389.65	0.54	0.4736	1598.29	1	1598.29	5.91	0.0281					
$a_{p}{}^{2}$	5243.15	1	5243.15	7.27	0.0166	65.03	1	65.03	0.24	0.6310					
Residual	10,813.15	15	720.88	—	—	4057.11	15	270.47	—	—	2.07	19	0.11	—	—
Lack of Fit	3676.14	10	367.61	4.86	0.0474	3676.14	10	367.61	4.82	0.0482	0.00	14	0.15	—	—
Pure Error	1008.30	5	210.66	—	—	380.97	5	76.19	—	—	813.89	5	0.00	—	—
Cor Total	153,800	29	—	—	—	52,151.54	29	—	—	—		29	—	—	—

**Table 6:** Verify the results of the experiment.

	1	2	3	Average	Predicted Value
*F*/(N)	208.81	221.69	193.75	208.08	234.81
*VB*/(μm)	29.67	30.84	27.22	29.24	33.40
*Q*/(mm^3^/min)	38.40	40.30	47.10	41.87	44.65

## Data Availability

Not applicable.
